# Extraction of elementary rate constants from global network analysis of *E. coli *central metabolism

**DOI:** 10.1186/1752-0509-2-41

**Published:** 2008-05-07

**Authors:** Jiao Zhao, Douglas Ridgway, Gordon Broderick, Andriy Kovalenko, Michael Ellison

**Affiliations:** 1 Institute for Biomolecular Design, University of Alberta, Edmonton, Alberta T6G 2H7, Canada; 2 Department of Medicine, University of Alberta, Edmonton, Alberta T6G 2H7, Canada; 3 NRC National Institute for Nanotechnology, Edmonton, Alberta T6G 2M9, Canada; 4 Department of Biochemistry, University of Alberta, Edmonton, Alberta T6G 2H7, Canada

## Abstract

**Background:**

As computational performance steadily increases, so does interest in extending one-particle-per-molecule models to larger physiological problems. Such models however require elementary rate constants to calculate time-dependent rate coefficients under physiological conditions. Unfortunately, even when *in vivo *kinetic data is available, it is often in the form of aggregated rate laws (ARL) that do not specify the required elementary rate constants corresponding to mass-action rate laws (MRL). There is therefore a need to develop a method which is capable of automatically transforming ARL kinetic information into more detailed MRL rate constants.

**Results:**

By incorporating proteomic data related to enzyme abundance into an MRL modelling framework, here we present an efficient method operating at a global network level for extracting elementary rate constants from experiment-based aggregated rate law (ARL) models. The method combines two techniques that can be used to overcome the difficult properties in parameterization. The first, a hybrid MRL/ARL modelling technique, is used to divide the parameter estimation problem into sub-problems, so that the parameters of the mass action rate laws for each enzyme are estimated in separate steps. This reduces the number of parameters that have to be optimized simultaneously. The second, a hybrid algebraic-numerical simulation and optimization approach, is used to render some rate constants identifiable, as well as to greatly narrow the bounds of the other rate constants that remain unidentifiable. This is done by incorporating equality constraints derived from the King-Altman and Cleland method into the simulated annealing algorithm. We apply these two techniques to estimate the rate constants of a model of *E. coli *glycolytic pathways. The simulation and statistical results show that our innovative method performs well in dealing with the issues of high computation cost, stiffness, local minima and uncertainty inherent with large-scale non-convex nonlinear MRL models.

**Conclusion:**

In short, this new hybrid method can ensure the proper solution of a challenging parameter estimation problem of nonlinear dynamic MRL systems, while keeping the computational effort reasonable. Moreover, the work provides us with some optimism that physiological models at the particle scale can be rooted on a firm foundation of parameters generated in the macroscopic regime on an experimental basis. Thus, the proposed method should have applications to multi-scale modelling of the real biological systems allowing for enzyme intermediates, stochastic and spatial effects inside a cell.

## Background

As systems biology matures, it is moving away from static representations of network interactions based on nodes and edges to dynamic representations that describe cellular processes in space and time. Dynamic metabolic processes are quantitatively modelled with ordinary differential equations (ODEs) in two principle ways: the aggregated rate law [1] and mass action rate law [2] approaches. These two models differ from one another in the level of detail at which they operate and hence the contexts in which they can be validly applied.

Dynamic metabolic networks are predominately modelled using aggregated rate laws (ARL). An ARL simplifies the description of a single enzymatic step by aggregating the elementary steps associated with a specific mechanism into a single reaction, where the rate becomes a non-linear function of substrate, product and regulator concentrations and a typically linear function of enzyme concentration. The classical example of an ARL treatment is the Michaelis-Menten equation for the simplest irreversible reaction, the Uni-Uni mechanism. ARL models are not always derived from an underlying mechanism, and in some cases, more phenomenological formulas are adopted to empirically fit experimental data. While the simplified treatment of enzyme kinetics using an ARL-based approach has obvious appeal when attempting to model large metabolic networks, such a reduction of the underlying mechanism inevitably leads to a loss of some useful information about the reaction mechanism, e.g. the sequence of elementary reactions, dynamics of all enzyme intermediates, and so on. ARL models are therefore equipped to capture biological processes on long time scales wherein dynamics of enzyme intermediates can be ignored. To date most experimentally determined kinetic parameters are at this level.

An alternative to the ARL approach is the direct use of mass action rate laws (MRL), each of which states that the rate of an elementary reaction is directly proportional to the product of the effective concentrations of each participating molecule. By definition, MRL models involve the sequence of elementary reactions as well as track dynamics of all of the elements by describing the formation and degradation of all species in an enzymatic reaction. It is particularly suitable for modeling molecular events that happen on the microsecond to millisecond time-scale [3,4].

Regardless of the approach taken, good models of cellular systems are often guided by a pragmatic principle: a model should be as simple as possible, but as complex as necessary. The growing necessity of dealing with complexity is however highlighted by the apparent behavioural differences exhibited by biomolecules within an intracellular environment versus the test tube [5]. These differences can be largely attributed to a range of spatial phenomena including macromolecular crowding, caging, spatial segregation of reactants, and the unpredictable nature associated with the reaction of rare and non-uniformly distributed biomolecules [6]. Significantly, a comprehensive quantitative understanding of most of these phenomena is lacking. Meanwhile, the steady increase in computational capability, coupled with improved technology for making quantitative measurements of single molecules within single living cells, is fuelling interest in an alternative modelling approach in which individual molecules are represented as particles that are imbued with the dynamic properties of movement and reaction as a function of space and time [7-9]. This approach, referred to as Particle Based Simulation (PBS), has the advantage that it can seamlessly link stochastic and continuous processes in a modelling environment where the spatial and physicochemical complications referred to above are represented explicitly. These explicit simulations require however the direct elementary rate constants and enzyme intermediates that distinguish MRL modelling from ARL modelling. If a system has been parameterized as an ARL model, it must first be converted into MRL form in order to set up a PBS simulation. Thus the MRL model becomes the bridge between the ARL format and the PBS format.

As a consequence of its higher level of detail, the MRL approach is starting to receive special attention for the elucidation of complex biological systems [10-14]. To date the biggest limitation associated with the MRL approach is the lack of detailed quantitative biochemical data to fuel the models [12]. This dearth of data has prompted the development of several estimation methods for the mass-action rate constants of enzymatic reactions.

The classic approach combines the Schematic Method of King and Altman [15] with the General Rule of Cleland [16] (SMKA/GRC). SMKA/GRC relates unknown elementary rate constants to known ARL kinetic constants. Individual rate constants are then calculated by solving linear or non-linear algebraic equations. One limitation of SMKA/GRC is that most of the experimentally determined ARL constants are derived from isolated enzymes *in vitro *over a range of conditions. This lack of biological context calls into question the relevance of network models based on these parameters in describing complex cellular behaviour under physiological conditions. One other limitation is that it is not always possible to uniquely determine the rate constants from existing ARL rate constants, when: 1) the number of MRL parameters is large, 2) there is a redundancy of values among ARL kinetic constants or 3) there are technical difficulties in solving nonlinear equations. Moreover, use of SMKA/GRC method alone is not able to deal with empirical ARL equations where some kinetic constants are missing or reduced to empirical constants.

Recently, Yang et al. [11] have proposed a simpler alternative to SMKA/GRC termed the Lambda and Omega approximation method. Estimating rate constants from available *in vitro *kinetic data (K_m_, K_cat _and K_i_) involves a rapid equilibrium approximation that assumes that reactants, free enzymes and enzyme-bound intermediates reach equilibrium quickly relative to the rate of catalysis. The approach to steady-state can be controlled in the model by using large, time-invariant constant numbers (**Λ **and **Ω**) that are associated with enzyme-substrate and enzyme-inhibitor binding reactions, respectively. When compared with SMKA/GRC, this approximation method is based on fewer kinetic constants and simpler algebraic relations, leading to easier mathematical manipulations. It does however suffer from limitations such as the existence of trimolecular association reactions that are physiologically improbable (e.g. the enzyme can interact simultaneously with two substrates in a Bi Bi mechanism) and the inherent ambiguity imposed by the dependence of the rate constants on arbitrary values of Λ and **Ω**.

The final method is numerical simulation and optimization (NSO) [17] for network models, where non-linear least squares regression is used in combination with simulation to optimize parameters from time-course variables. In principle, this method could be used to find optimal MRL rate constants provided that enough time-course data and constraints are available. However, this strategy often meets with difficulties because even relatively simple metabolic pathways modelled by MRL expand to large and stiff systems of ODEs. Unsurprisingly then, the use of NSO in MRL modelling is not prevalent and is often constrained to the analysis of small systems [17]. Furthermore, the complexity of the parameter space coupled with poor knowledge of the *in vivo *rate constants means that the optimization algorithm is easily trapped by local minima [18] or returns a family of solutions [19]. By comparison, for ARL models, NSO has been successfully applied to globally optimize the parameters for more complex metabolic networks [20,21]. If a method could be developed that deals with the issues of network scale, stiffness, local minima and parameter identifiability, then NSO could play an important role in the development of detailed, complex and therefore useful MRL models.

In this work, we present a novel methodology for extracting MRL elementary rate constants from ARL network models, which combines the advantages of two techniques with respect to model structure. Our ultimate goal is to generate an automatic transformation from ARL kinetic information into the elementary MRL rate constants required for our PBS modelling effort. When it is applied to a challenging parameterization problem in regards to central metabolism of *E. coli*, our method proved efficient and robust, thereby enabling systemic investigation of the mass action rate laws of a large-scale cellular network.

## Results

### Method evaluation

**Figure 1:**
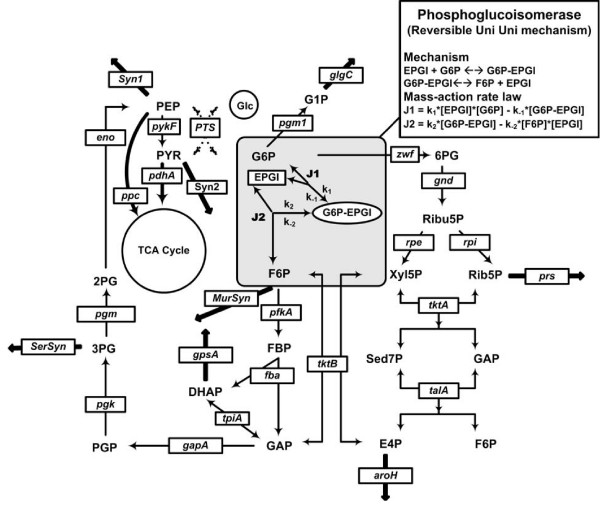
**Reactions and topology of the *E. coli *central metabolism as used in the ARL model**. As an example, the *pgi *pathway described as aggregated rate law (ARL) was replaced by mass-action rate law (MRL) for estimation of the elementary rate constants. The same procedure can be used to estimate rate constants involved in other pathways.

In parameter estimation, the principle issues are the precision of the estimates and the practical reality of the computational burden. This paper presents and assesses the new method, in terms of computational cost, parameter identifiability and the effect of relative uncertainty in measured data. Evaluation was conducted by setting the glycolytic pathways of *E. coli *to mass action kinetics and adjusting rate constants to result in same flux and concentration dynamics as the original ARL model [21]. The MRL network is approximately three times larger than its ARL analogue, owing to the expansion of individual ARL steps into their mechanistic sub-steps. Because of this added level of complexity, only a single set of MRL steps (*pgi*) is shown within its ARL context (Figure 1) for illustrative purposes, with the full MRL decomposition presented in Table 1. The optimized parameters and the statistical analysis of the results are summarized in Table 2.

**Table 1:** Reaction mechanism and protein abundance for glycolytic pathways of *E. coli*

**Enzyme**	**Abundance ^a ^(molecules/cell)**	**Concentration ^b ^(μM)**	**Kinetic mechanism**	**Elementary reactions**
Phosphogluco -isomerase *(pgi)*	1099	2.7	Reversible Uni Uni [51]	G6P + EPGI → EPGI-G6P
				EPGI-G6P → G6P + EPGI
				EPGI-G6P → F6P +EPGI
				F6P + EPGI → EPGI-G6P
Phosphofructo -kinase *(pfkA)*	3287	8.2	Allosteric regulation and ordered sequential mechanism [39] [52]	ATP + EPFKA_TR _→ EPFKA_R_-ATP
				EPFKA_R_-ATP → ATP + EPFKA_TR_
				F6P + EPFKA_R_-ATP → EPFKA_R_-ATP-F6P
				EPFKA_R_-ATP-F6P → F6P + EPFKA_R_-ATP
				EPFKA_R_-ATP-F6P → FDP + EPFKA_R_-ADP
				EPFKA_R_-ADP → ADP + EPFKA_TR_
Fructose-bisphosphate aldolase *(fba)*	16326	40.5	Ordered Uni Bi [51]	FDP + EFBA → EFBA-FDP
				EFBA-FDP → FDP + EFBA
				EFBA-FDP → GAP + EFBA-DHAP
				GAP + EFBA-DHAP → EFBA-FDP
				EFBA-DHAP → DHAP + EFBA
				DHAP + EFBA → EFBA-DHAP
Triosephosphate isomerase *(tpiA)*	9106	22.6	Reversible Uni Uni [51]	DHAP + ETPIA → ETPIA-DHAP
				ETPIA-DHAP → DHAP + ETPIA
				ETPIA-DHAP → GAP + ETPIA
				GAP + ETPIA → ETPIA-DHAP
Glyceraldehyde 3-phosphate dehydrogenase *(gapA)*	49091	121.7	Ordered sequential mechanism [37]	NAD + EGAP → EGAP-NAD
				EGAP-NAD → NAD + EGAP
				GAP + EGAP-NAD → EGAP-NAD-GAP
				EGAP-NAD-GAP → GAP + EGAP-NAD
				EGAP-NAD-GAP → PGP + EGAP-NADH
				PGP + EGAP-NADH → EGAP-NAD-GAP
				EGAP-NADH → NADH + EGAP
				NADH + EGAP → EGAP-NADH
Phosphoglycerate kinase *(pgk)*	14682	36.4	Ordered sequential mechanism [53]	PGP + EPGK → EPGK-PGP
				EPGK-PGP → PGP + EPGK
				ADP + EPGK-PGP → EPGK-PGP-ADP
				EPGK-PGP-ADP → ADP + EPGK-PGP
				EPGK-PGP-ADP → ATP + EPGK-PG3
				ATP + EPGK-PG3 → EPGK-PGP-ADP
				EPGK-PG3 → PG3 + EPGK
				PG3 + EPGK → EPGK-PG3
Phosphoglycerate mutase *(pgm)*	966	2.4	Reversible Uni Uni [21]	PG3 + EPGM → EPGM-PG3
				EPGM-PG3 → PG3 + EPGM
				EPGM-PG3 → PG2 + EPGM
				PG2 + EPGM → EPGM-PG3
Enolase *(eno)*	11283	28.0	Reversible Uni Uni [21]	PG2 + EENO → EENO-PG2
				EENO-PG2 → PG2 + EENO
				EENO-PG2 → PEP + EENO
				PEP + EENO → EENO-PG2
Pyruvate kinase *(pykF) *^b^	500	1.2	Allosteric regulation and ordered sequential mechanism [24, 39]	PEP + EPYKF_TR _→ EPYKF_R_-PEP
				EPYKF_R_-PEP → PEP + EPYKF_TR_
				EPYKF_R_-PEP + ADP → EPYKF_R_-PEP-ADP
				EPYKF_R_-PEP-ADP → EPYKF_R_-PEP + ADP
				EPYKF_R_-PEP-ADP → PYR + EPYKF_R_-ATP
				EPYKF_R_-ATP → ATP + EPYKF_TR_

^a ^Protein abundances are taken from mass spectrometry data [34], with the exception of the abundance for *pykF *which is taken from 2-D gel data [35]

^b ^Volume concentration is calculated based on *E. coli *cytoplasm volume equivalent to 6.7 × 10^-16 ^L [54]

**Table 2:** Summary statistics about rate constants estimated from the proposed hybrid method ^a,b^

**Enzyme**	**Ordinary differential equations**	***K*-values ^c ^(Mean ± SD)**	**CV (%)**	**CI (95%)**	***fval*-values (Mean ± SD)**
Phosphogluco -isomerase *(pgi)*	K_1f,PGI_*[G6P]*[EPGI]	(1.27 ± 0.00) × 10^5^	0.0	(1.27, 1.27) × 10^5^	(9.2 ± 2.4) × 10^-3^
	K_1r,PGI_*[EPGI-G6P]	(1.28 ± 0.00) × 10^5^	0.0	(1.28, 1.28) × 10^5^	
	K_2f,PGI_*[EPGI-G6P]	(2.41 ± 0.00) × 10^5^	0.0	(2.41, 2.41) × 10^5^	
	K_2r,PGI_*[F6P]*[EPGI]	(1.39 ± 0.01) × 10^6^	0.0	(1.39, 1.39) × 10^6^	
Phosphofructo -kinase *(pfkA)*^d^	K_1f,PFKA_*f*[EPFKA_TR_]*[ATP]^m^	(4.21 ± 0.63) × 10^1^	15.0	(3.83, 4.59) × 10^1^	(5.4 ± 1.2) × 10^-2^
	K_1r,PFKA_*[EPFKA_R_-ATP]	(1.73 ± 1.94) × 10^0^	112.7	(0.55, 2.90) × 10^0^	
	K_2f,PFKA_*[EPFKA_R_-ATP]*[F6P]^n^	(4.70 ± 0.50) × 10^3^	10.7	(4.39, 5.00) × 10^3^	
	K_2r,PFKA_*[EPFKA_R_-ATP-F6P]	(6.52 ± 7.93) × 10^1^	121.6	(1.73, 11.31)×10^1^	
	K_3f,PFKA_*[EPFKA_R_-ATP-F6P]	(3.50 ± 0.76) × 10^2^	21.8	(3.05, 3.96) × 10^2^	
	K_4f,PFKA_*[EPFKA_R_-ADP]	(3.67 ± 3.01) × 10^5^	82.2	(1.85, 5.49) × 10^5^	
Fructose- bisphosphate aldolase *(fba)*	K_1f,FBA_*[FDP]*[EFBA]	(4.98 ± 0.01) × 10^3^	0.2	(4.98, 4.98) × 10^3^	(4.8 ± 0.1) × 10^-3^
	K_1r,FBA_*[EFBA-FDP]	(9.14 ± 0.02) × 10^2^	0.2	(9.13, 9.14) × 10^2^	
	K_2f,FBA_*[EFBA-FDP]	(4.33 ± 0.00) × 10^2^	0.0	(4.33, 4.33) × 10^2^	
	K_2r,FBA_*[GAP]*[EFBA-DHAP]	(9.98 ± 0.02) × 10^4^	0.2	(9.98, 9.99) × 10^4^	
	K_3f,FBA_*[EFBA-DHAP]	(5.95 ± 0.01) × 10^4^	0.2	(5.94, 5.95) × 10^4^	
	K_3r,FBA_*[DHAP]*[EFBA]	(1.04 ± 0.00) × 10^4^	0.0	(1.04, 1.04) × 10^4^	
Triosephosphate isomerase *(tpiA)*	K_1f,TPIA_*[DHAP]*[ETPIA]	(1.17 ± 0.00) × 10^3^	0.0	(1.17, 1.17) × 10^3^	(1.5 ± 0.6) × 10^-4^
	K_1r,TPIA_*[ETPIA-DHAP]	(2.34 ± 0.00) × 10^2^	0.0	(2.34, 2.34) × 10^2^	
	K_2f,TPIA_*[ETPIA-DHAP]	(3.04 ± 0.00) × 10^3^	0.0	(3.04, 3.04) × 10^3^	
	K_2r,TPIA_*[GAP]*[ETPIA]	(1.09 ± 0.00) × 10^4^	0.0	(1.09, 1.09) × 10^4^	
Glyceraldehyde 3- phosphate dehydrogenase *(gapA)*	K_1f,GAP_*[NAD]*[EGAP]	(3.01 ± 0.00) × 10^4^	0.0	(3.01, 3.01) × 10^4^	(7.8 ± 0.4) × 10^-2^
	K_1r,GAP_*[EGAP-NAD]	(7.57 ± 0.00) × 10^3^	0.0	(7.57, 7.57) × 10^3^	
	K_2f,GAP_*[GAP]*[EGAP-NAD]	(1.85 ± 0.25) × 10^4^	13.5	(1.75, 1.95) × 10^4^	
	K_2r,GAP_*[EGAP-NAD-GAP]	(9.61 ± 0.90) × 10^5^	9.4	(9.25, 9.96) × 10^5^	
	K_3f,GAP_*[EGAP-NAD-GAP]	(1.67 ± 0.83) × 10^6^	50.0	(1.34, 2.00) × 10^6^	
	K_3r,GAP_*[PGP]*[EGAP-NADH]	(1.99 ± 0.64) × 10^9^	32.3	(1.74, 2.24) × 10^9^	
	K_4f,GAP_*[EGAP-NADH]	(7.61 ± 0.01) × 10^3^	0.2	(7.61, 7.62) × 10^3^	
	K_4r,GAP_*[NADH]*[EGAP]	(7.36 ± 0.13) × 10^-1^	0.2	(7.35, 7.37) × 10^-1^	
Phosphoglycerate kinase *(pgk)*	K_1f,PGK_*[PGP]*[EPGK]	(1.77 ± 0.00) × 10^6^	0.0	(1.77, 1.77) × 10^6^	(3.4 ± 1.4) × 10^-3^
	K_1r,PGK_*[EPGK-PGP]	(8.30 ± 0.00) × 10^4^	0.0	(8.30, 8.30) × 10^4^	
	K_2f,PGK_*[ADP]*[EPGK-PGP]	(4.58 ± 0.05) × 10^5^	0.0	(4.57, 4.60) × 10^5^	
	K_2r,PGK_*[EPGK-PGP-ADP]	(4.52 ± 0.35) × 10^3^	7.8	(4.41, 4.63) × 10^3^	
	K_3f,PGK_*[EPGK-PGP-ADP]	(3.47 ± 3.45) × 10^5^	99.5	(2.36, 4.57) × 10^5^	
	K_3r,PGK_*[ATP]*[EPGK-PG3]	(5.10 ± 4.99) × 10^5^	97.9	(3.50, 6.69) × 10^5^	
	K_4f,PGK_*[EPGK-PG3]	(1.64 ± 0.88) × 10^5^	53.6	(1.36, 1.92) × 10^5^	
	K_4r,PGK_*[PG3]*[EPGK]	(1.24 ± 0.68) × 10^5^	54.7	(1.03, 1.46) × 10^5^	
Phosphoglycerate Mutase *(pgm)*	K_1f,PGM_*[PG3]*[EPGM]	(2.01 ± 0.00) × 10^6^	0.0	(2.01, 2.01) × 10^6^	(2.8 ± 1.3) × 10^-2^
	K_1r,PGM_*[EPGM-PG3]	(3.64 ± 0.00) × 10^5^	0.0	(3.64, 3.64) × 10^5^	
	K_2f,PGM_*[EPGM-PG3]	(3.71 ± 0.00) × 10^4^	0.0	(3.71, 3.71) × 10^4^	
	K_2r,PGM_*[PG2]*[EPGM]	(1.08 ± 0.01) × 10^6^	1.3	(1.07, 1.09) × 10^6^	
Enolase *(eno)*	K_1f,ENO_*[PG2]*[EENO]	(1.42 ± 0.00) × 10^5^	0.0	(1.42, 1.42) × 10^5^	(2.6 ± 1.2) × 10^-2^
	K_1r,ENO_*[EENO-PG2]	(2.37 ± 0.00) × 10^3^	0.0	(2.37, 2.37) × 10^3^	
	K_2f,ENO_*[EENO-PG2]	(1.18 ± 0.00) × 10^4^	0.0	(1.18, 1.18) × 10^4^	
	K_2r,ENO_*[PEP]*EENO]	(1.05 ± 0.02) × 10^5^	1.9	(1.05, 1.06) × 10^5^	
Pyruvate kinase *(pykF) *^e^	K_1f,PYKF_*f*[EPYKF_TR_]*[PEP]	(1.59 ± 0.00) × 10^2^	0.0	(1.59, 1.59) × 10^2^	(6.0 ± 2.0) × 10^-4^
	K_1r,PYKF_*[EPYKF_R_-PEP]	(4.96 ± 0.17) × 10^1^	3.4	(4.89, 5.03) × 10^1^	
	K_2f,PYKF_*[EPYKF_R_-PEP]*[ADP]	(4.58 ± 2.92) × 10^2^	63.9	(3.37, 5.78) × 10^2^	
	K_2r,PYKF_*[EPYKF_R_-PEP-ADP]	(2.43 ± 1.85) × 10^2^	76.2	(1.67, 3.19) × 10^2^	
	K_3f,PYKF_*[EPYKF_R_-PEP-ADP]	(2.71 ± 1.71) × 10^2^	63.2	(2.00, 3.41) × 10^2^	
	K_4f,PYKF_*[EPYKF_R_-ATP]	(7.89 ± 3.09) × 10^1^	39.2	(6.61, 9.16) × 10^1^	

^a ^Rate constants are the statistical solutions from 25 optimization runs which were performed with different random initial guesses for parameters of an individual enzyme. Through hybrid MRL/ARL model, MRL parameters for each enzyme are estimated in separate steps. Units are mM^-1 ^s^-1 ^and s^-1 ^for second and first-order rate constants, respectively.

^b ^SD: Standard deviation; CV: Coefficient of variation; CI: Confidence interval; *fval*: cost function value.

^c ^Due to uncertainty in the source data [21], we have truncated to three digits, regarding of SD.

^d ^Reaction orders for allosteric enzyme *pfkA *are non-integer with respect to ATP and F6P, allowing for fractal properties [43] due to enzyme conformational changes. Values of m and n are 5.19 ± 0.28 and 9.80 ± 0.41, respectively, after optimization.

^e ^Reaction for allosteric enzyme pyruvate kinase is first order with respect to PEP and ADP, according to Tormonia's paper [39]

#### Computational cost

Given an optimization algorithm, one of the challenges for a large-scale nonlinear model is the computational economy of that method. This includes how to deal with large numbers of parameters and how to circumvent bottlenecks that limit algorithm performance. These issues must be addressed to make parameter estimation a practical reality; otherwise the computational effort may go beyond a reasonable amount of time for MRL models [22].

The principle technique applied here is a hybrid ARL/MRL strategy, which reduces the number of parameters that need to be estimated simultaneously. This technique allows us to estimate parameters of the mass action rate laws for each enzyme in separate steps. In addition, the number of free parameters is further reduced by adding equality constraints derived from algebraic SMKA/GRC method. However, the global optimization still consumes substantial computational time, since it requires vast numerical integrations of ODEs in order to evaluate the cost function at each iteration step. Particularly, the computational cost increases further when the target model has the significant stiffness that often appears in mass action equations for enzymatic reaction systems. In such systems, each solution requires small integration steps to accommodate the introduction of fast-varying enzyme intermediates. By converting Matlab M-files for differential equations into MEX-files (MEX stands for MATLAB Executable files, which are dynamically linked subroutines produced from C source code), as well as by opting for the stiff ode15s solver, up to 10-fold faster optimization can be achieved. Each optimization run was able to quickly obtain optimal solutions within several minutes with the computer environment as follows: Intel Core™ 2 Duo Processor 1.73 GHz CPU with memory size of 2.5 GB, thereby facilitating the repetition of the optimization many times for statistical purposes.

#### Identifiability

Other challenges for optimizing a large-scale nonlinear model include local minima and non-convex regions over the objective function space. In this work, we adopted the simulated annealing algorithm, which combines the advantages of our two proposed techniques (i.e. hybrid MRL/ARL modeling and hybrid algebraic-numerical optimization), for globally minimizing multivariate functions.

To ensure that the algorithm is not trapped in sub-optimal local minima within a large search space, a suitable number of optimizations (25 runs) were done with different random initial guesses over the entire range of the rate constants. Our cost function values (*fval*) show that the method is able to consistently return parameters from which the MRL model dynamics closely matched those observed for the original ARL model, thereby avoiding the local minima problem leading to a badness of fit between ARL and MRL dynamics.

It has been found that the candidate values of the rate constants may be highly correlated [12] and the search surface may consist of a very flat valley floor [23], resulting in unreliable or unreproducible estimates although the fit of model to data may be very good. Such ill-posed/non-convex optimization problems must be taken into account while assessing the quality of MRL model fit to ARL data. The coefficient of variation (CV), defined as the standard deviation divided by the mean, was used as a measure of the reproducibility of the results from 25 optimization runs. Distributions with a CV < 10% are considered high-precision and low-variance, while those with a CV varying between 10% and 50% are considered moderate precision and variance. Table 2 shows that CV for 80% of parameters was below 50%, with 66% of parameters with CV < 10% and with 14% of parameters with CV in the range of 10 – 50%, indicating that agreement between the optimization runs varied from moderate to very good for most parameters. Estimates of 20% of parameters are associated with CV ranging from 54% to 122%, which suggests that these parameters are not highly identifiable from the existing kinetic information. Nevertheless, the confidence interval shows that with 95 percent certainty the actual values for these unidentifiable rate constants fall within a much narrower range than that for the original biological bounds. Apparently, these unidentifiable parameters are interval-identifiable, being bounded within a finite interval from the existing ARL dynamics, algebraic equality and inequality constraints.

**Figure 2:**
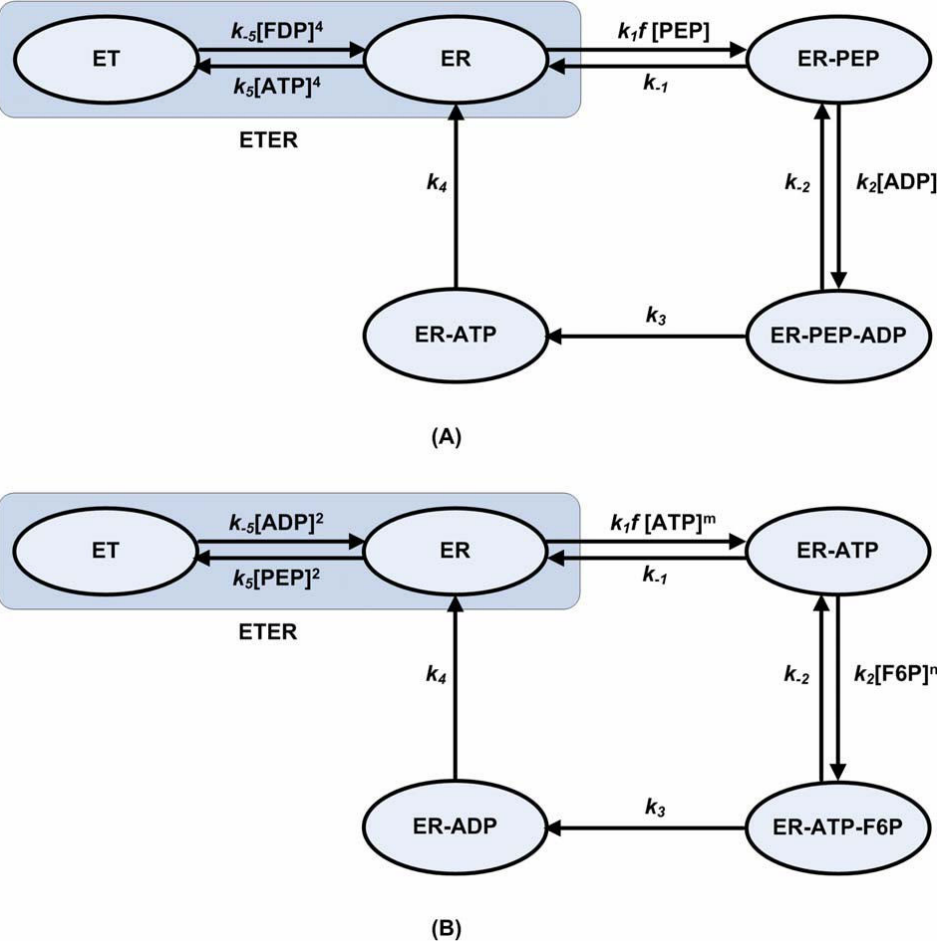
**Allosteric enzymes consisting of an allosteric segment (dark shaded area) and an enzymatic reaction segment**. (A): Pyruvate kinase, where the number of allosteric sites and catalytic sites are 4 and 1, respectively [39]. (B): Phosphofructokinase, where the number of allosteric sites is 2 according to [39]. Non-integral reaction orders are assumed for two substrates due to fractal properties caused by enzyme conformational changes [43].

Note that in particular cases, some biological restrictions applied to *k *values during optimization enable the proposed method to constrain the range of values permitted for some unknown parameters which are otherwise not determinable. The *pykF *pathway is an example in which the ARL kinetic information is not adequate to fully specify the underlying rate constants, leaving up to three undetermined rate constants for the proposed mechanism coupling allosteric regulation with sequential Bi Bi reactions (Figure 2A). Experimental results have already shown that direct phosphoryl transfer takes place in the ternary complex [24], thereby excluding the Theorell-Chance Bi-Bi mechanism, where only binary complexes are formed. This experimental evidence provides strong inequality constraints on the rate constants *k*_-2 _and *k*_3_: they cannot be very large compared to *k*_-1 _and *k*_4_, respectively; otherwise no stable ternary complex can be formed. Consequently, we include a constraint on *k*_-2 _to ensure that it is less than 10*k_-1_. The value of *k*_-1_, on the other hand, always converges to a solution around 50 s^-1 ^after optimization regardless of boundary condition. So we set an upper bound of 500 s^-1 ^for *k*_-2_. Similarly, the value of the other adjustable parameter, *k*_3_, is constrained to be less than 10*k_4_. This constraint enables *k*_3 _to fall within the region of 50 to 500 s^-1 ^after optimization. Apparently, these biological inequality restrictions can assistant our method to narrow the bounds of some rate constants that remain unidentifiable.

#### Experimental uncertainty

Another important issue in the parameter estimation process is the existence of uncertainty in the experimental data, including both the concentration time courses and the enzyme abundances used in our method. These uncertainties can affect the parameter estimates, especially since the enzyme concentration for *pykF *has been obtained through analysis of 2-D gels. Such gel-based proteome technique is frequently subject to gel-to-gel variations [25], so it is more susceptible to noise as compared with other measurement techniques. Because of potential high noise levels in analysis of 2-D gels, we use *pykF *as a representative best case to investigate the bias and variation of parameter estimation caused by uncertainty in experimental measurements. We added Gaussian distributed random variates to the experimentally determined value for *pykF *(1.2 μM, which is taken as the 'noise-free' value in this work). 25 such noisy enzyme concentrations were generated for each of two noise levels (10% and 20%). A 95% confidence interval (CI) for the fitted parameters and the relative errors (RE) between noise and noise-free solutions were used to evaluate the precision and bias due to experimental uncertainty of enzyme level.

Table 3 shows that the order-of-magnitudes of rate constants are not affected by adding noise to the experimental measured data. Moreover, the 95% confidence intervals fall within a relatively narrow range almost independently of the test noise. As far as RE is concerned, the system response is different when comparing between 10% and 20% noise level. It appears that RE increases with the noise level, with the exception of k_2r _and k_3f_. Despite an increase, most RE can be kept at low level. With 10% noise, RE for 4 out of 6 parameters was below 10%, while with 20% noise RE for 5 out of 6 parameters falls within 10%. These results suggest that the parameter estimates are relatively insensitive to noise below a certain level. However, the parameter certainty deteriorated when the level of noise was raised to 30%. RE for some rate constants were over 50% and the 95% confidence interval was significantly expanded (data not shown).

**Table 3:** Effect of uncertainty in *pykF *enzyme concentration measurement on rate constants estimation ^a^

**Parameters**	**0% noise ^b^**			**10% noise ^c^**			**20% noise ^c^**		
			
	**Mean ± SD × 10^2^**	**95% CI × 10^2^**	**RE %**	**Mean ± SD × 10^2^**	**95% CI × 10^2^**	**RE %**	**Mean ± SD × 10^2^**	**95% CI × 10^2^**	**RE %**
**K_1f_**	1.59 ± 0.00	[1.59, 1.59]	0	1.61 ± 0.17	[1.54, 1.68]	1.3	1.51 ± 0.21	[1.42, 1.60]	5.0
**K_1r_**	0.50 ± 0.02	[0.49, 0.50]	0	0.50 ± 0.06	[0.47, 0.52]	0	0.47 ± 0.07	[0.44, 0.50]	6.0
**K_2f_**	4.58 ± 2.92	[3.37, 5.78]	0	4.61 ± 3.76	[3.05, 6.16]	0.7	4.52 ± 2.47	[3.50, 5.54]	1.3
**K_2r_**	2.43 ± 1.85	[1.67, 3.19]	0	1.96 ± 1.76	[1.24, 2.69]	19.3	2.62 ± 1.71	[1.90, 3.32]	7.8
**K_3f_**	2.71 ± 1.71	[2.00, 3.41]	0	2.69 ± 1.63	[2.02, 3.36]	0.7	2.70 ± 1.51	[2.08, 3.32]	0.4
**K_4f_**	0.79 ± 0.31	[0.66, 0.92]	0	0.89 ± 0.56	[0.66, 1.12]	12.7	0.66 ± 0.19	[0.58, 0.74]	16.5

^a ^SD: Standard deviation; CI: Confidence interval; RE: Relative errors compared to 0% noise case.

^b ^Rate constants are the statistical solutions from 25 optimization runs which were performed with random initial guesses for parameters of *pykF*.

^c ^Rate constants are the statistical solutions from 25 optimization runs which were performed with random initial guesses for parameters and with Gaussian noise added to the experimentally determined *pykF *concentration.

In short, our results indicate that the proposed method can be applied to moderately noisy data. In particular, we have shown for the *pykF *example the modest impact on parameter estimation for an underlying MRL model at a 20% uncertainty in enzyme level. For proteins with a dramatically high uncertainty from 2-D gel analysis, several techniques, such as prefractionation, parallel and repeated run of gels, are available to reduce the noise level before these proteomic data are incorporated in our method.

### Model evaluation

#### Parameter sensitivity

We then investigated how these optimal parameters influenced the systemic response, which are normally quantified through sensitivity analysis using the methods of Metabolic Control Analysis (MCA) at steady state. Our interest, however, was to examine the effect of changing these parameters on the MRL system's temporal response, where the behaviour of interest is often found. We have therefore focused on time-dependent sensitivity analysis.

**Figure 3:**
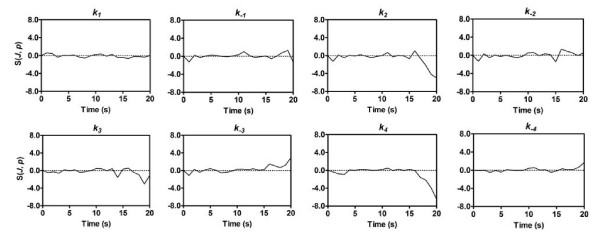
**Sensitivity analysis of the flux through glycolysis with respect to rate constants along *gapA *pathway**. See Figure 4A for the reaction mechanism of *gapA *pathway.

**Figure 4:**
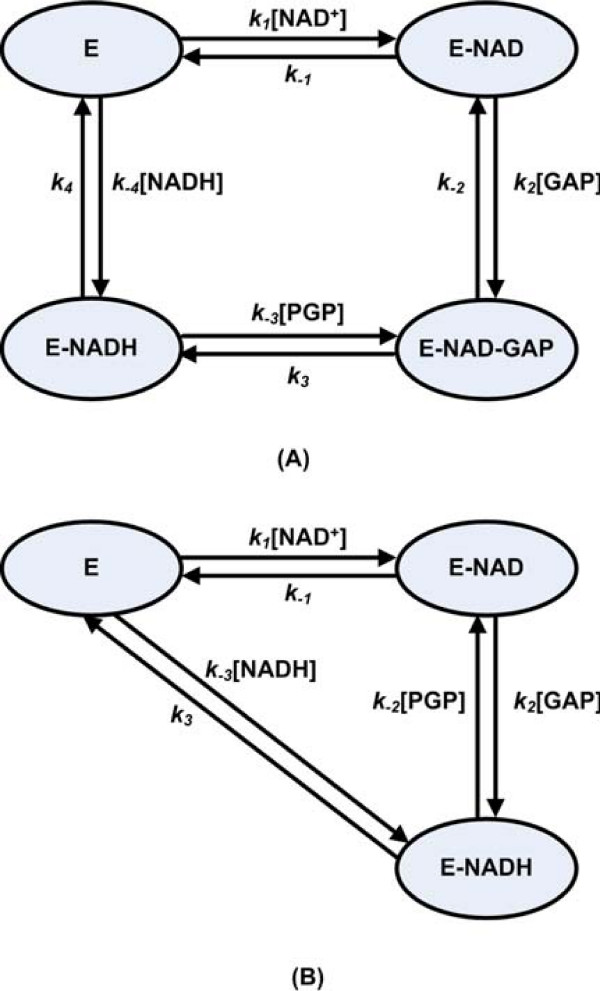
**Alternative reaction mechanisms of the Glyceraldehyde 3-phosphate dehydrogenase (*gapA*)**. A: Ordered Bi-Bi system with stable central ternary complex. B: Theorell-Chance Bi-Bi system with only the formation of stable binary complex.

We performed time-dependent sensitivity analysis of the flux of glucose through glycolysis (*J*_*PGI*_) with respect to rate constants along the glycolytic chain. The results are large time-varying matrices, which needs to be properly visualized. We present here the visualization results of sample analyses using time-dependent sensitivities for *gapA *pathway (Figure 3). The large sensitivity of the glycolytic response with respect to *gapA *parameters is found in the late portion of the transition window. The sensitivity analysis indicates, however, that a balance exists before 13 s where small increases or decreases in parameters have little effect on the glycolytic rate. The sensitivity with respect to the formation and breakdown of E-NAD-GAP (*k*_2_, *k*_-3 _and *k*_3_, see mechanism in Figure 4A) becomes significant after 15 s, indicating that the time-course of the system is highly dependent on the ternary complex. These results are very interesting, because they further emphasize the importance of the ternary complex for *gapA *rather than mere binary complexes. Also of interest is the dissociation step involving the last release of NAD^+ ^from the enzyme, resulting in a high sensitivity to the parameter *k*_4_.

#### Model performance

Since the rate constants for each enzyme are estimated in separate steps, the next question of interest would be what happens when these individual MRL parts are assembled together to form a coupled enzymatic reaction system. We therefore assembled these MRL parts into Chassagnole's central metabolism model to check functioning of the new assembly under actual operating conditions.

**Figure 5:**
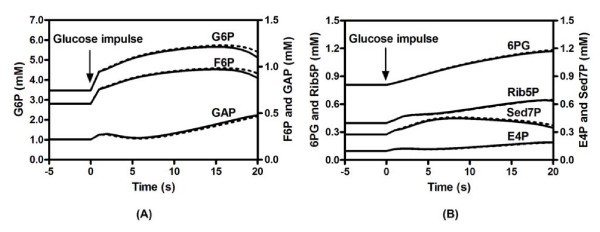
**Simulated dynamics of metabolites interacting between glycolysis and pentose phosphate pathway (PPP)**. A: glycolytic metabolites. B: PPP metabolites. MRL simulation: dotted line; ARL simulation: solid line. The time period over which we run the simulation is consistent with real experiments wherein all the intracellular metabolites could be sampled and measured within 20 seconds after glucose impulse [21].

The time-courses of some typical metabolites representing links between the pentose phosphate cycle and glycolysis are shown in Figure 5. The dynamic behaviour observed from the coupled MRL reaction system matched its ARL counterpart well in response to a glucose impulse, indicating that the MRL system can successfully replace the ARL system to represent the time-course data in the macroscopic regime. More importantly, the MRL system presents an opportunity to understand how an enzymatic reaction works by probing the elementary steps.

To compare the relative stabilities of the ARL and MRL networks, we first computed the Jacobian matrix to determine eigenvalues for both systems at steady-state. The largest MRL eigenvalue observed was -0.00079 s^-1^, and the spectrum of ARL values indicated a maximum of -0.00092 s^-1^. The fact that all eigenvalues prove to be negative for both models indicates that the ARL and MRL models are able to return to equilibrium following small perturbations. Since the MRL form essentially introduces fast variables that have been eliminated in the ARL form, it is not surprising that the MRL model greatly increases the eigenvalue spread, changing the smallest eigenvalue from -3.4 × 10^3 ^s^-1 ^to -1.9 × 10^7 ^s^-1^and the smallest time constant from 2.9 × 10^-1 ^ms to 5.3 × 10^-5 ^ms.

As a result of the ensuing introduction of enzyme forms to the model, the time scale range of reaction is greatly expanded by many orders of magnitude, rendering the MRL model considerably stiffer than its ARL counterpart (ARL stiffness- 3.7 × 10^6^, MRL stiffness- 2.4 × 10^10^). These widely differing time scales means that the usual numerical methods require small time step sizes to achieve stable solutions. For the metabolic network described here, we initially opted for Matlab's built-in forward differencing integrator, ode45, which failed to achieve solutions through its selection of inordinately small time steps for the model with both fast and slow changing variables. Therefore, we used the backward differencing solver ode15s to accommodate the inherent stiffness of the MRL model. Using this approach, the computational cost of an MRL simulation was still 33% larger than an ARL simulation. However, by compiling the MRL model into MEX binaries, the CPU time was reduced 87%.

## Discussion

The immediate motivation for our MRL modelling is to provide association and dissociation rate constants for the particle-based (PB) modelling aimed at building biophysical realism through four-dimensional simulations of *in vivo *elemental reactions. The incorporation of rate constants into PB modelling can be implemented by using the standard Smoluchowski theory [26] for computing time-dependent reaction coefficients and survival probability [27-29]. Due to the lack of high-quality experimentally determined rate constants, researchers have to make many, often arbitrary, assumptions on the values of these parameters, making this type of model less appealing to biologists. To the best of our knowledge, the method we present in this paper is the first step which allows passing *in vivo *data on an experimental basis to the dynamic multidimensional modelling at a finer scale. The work presented here provides us with some optimism that models operating at different scales can in fact be linked in a meaningful way.

In a more general sense, it is clear that increasingly sophisticated and reliable models of system dynamics will depend upon a sufficient underlying layer of biophysical detail so that they can respond and adapt realistically to changes in the physiological environment. Notably the ARL approach is capable of dealing with large networks by ignoring the details of enzyme intermediates and the rate constants that underpin biophysical reality. By comparison, MRL models provide the detailed framework required for a foundation for building biophysical realism. Thus, there is a distinct need for developing mechanistic MRL models which can provide more realistic predictions of cellular components and dynamics in a model organism.

MRL models belong to the class of non-convex nonlinear models wherein a number of difficulties may arise when estimating parameters of a realistic dynamical system, like e.g. convergence to local solutions, flat objective function in the neighbourhood of the solution and unreasonable computational effort for a problem with a large number of parameters. While previously most research work in this area has focused on the search algorithms (e.g. the hybrid stochastic-deterministic search [30], scatter search [23] and modern evolution search [17]), our work, however, focuses on the other side of the problem of parameter estimation, i.e. the model structure. We exploit the model structure to improve the efficiency and robustness of parameterizing a large-scale MRL model. It is based upon a strategy that identifiable structures or submodels can be generated by systematically eliminating parameters of the original model until it becomes identifiable [19]. We present a novel methodology with respect to parameter elimination without changing the original dynamics, which combines the advantages of two hybrid techniques. By replacing a single ARL pathway with its MRL equivalent, and installing this module to the same place as before while keeping other original ARL pathways unchanged, each set of MRL reactions can be independently and efficiently optimized. The alternative is an extremely cumbersome optimization process involving the simultaneous adjustment of an unreasonably large number of parameters. The model structure can be further manipulated by applying equality constraints to the rate constants associated to each enzyme. The resulting technique for incorporating parameter equality constraints into numerical simulation and optimization consistently reduces the number of parameters for a single enzyme, thereby ensuring maximum efficiency and robustness of the parameterization method. Consequently, our method may pave the way towards future systemic investigation of the mass action rate laws of large-scale cell network from widely accessible ARL models.

A problem that attracts continuing interest is that not all parameters in a large-scale non-convex nonlinear model are uniquely identifiable. DiStefano [31] introduced the notion of interval identifiabilty to describe finite bounds on the unidentifiable rate constants of general mammillary models. Vicini et al. [19] used additional parameter knowledge to narrow the bounds of rate constants that remain unidentifiable in mammillary and catenary compartmental models. In MRL models with respect to glycolysis of *E. coli*, we also found that 20% of parameters are not highly identifiable from the existing dynamics data. We incorporate several levels of coupling, including equality and inequality constraints and global optimization algorithm, to successfully reduce the range of computable bounds for highly unidentifiable parameters. Comparing with a wide-varying range applicable to mass-action rate constants, the range shrinking applied here is the best way so far to acquire reasonable approximations of the parameters.

In addition to their immediate motivational value for the particle-based modelling, our novel methodology and the resulting MRL models have some other interesting applications. For example, starting from the steady state before glucose impulse, the initial concentration for every enzyme form can be derived from the Schematic Method of King and Altman (SMKA). Then all enzyme forms freely evolve to comply with systemic dynamics under the constraint of fixed total concentration, thereby releasing the constraint of the widely-used quasi-steady state assumption (QSSA). This avoidance of QSSA will greatly extend the application area of the proposed method, since QSSA can be problematic for some *in vivo *pathways at high enzyme levels [32] and also for fast transient change reactions such as signalling and transduction pathways [14]. Parameter sensitivity is another important aspect that may be applicable to experiments regarding parameter identifiability. Through the time-dependent sensitivity analysis, parameters within a certain period of time demonstrate little impact on the simulator results (Figure 3). It is therefore not worthwhile focusing experiments on this period to tune the parameters. Moreover, sensitivity analysis reveals key elementary reaction steps that would affect the overall dynamics of the metabolic network. One potential approach to accelerate optimization convergence would be to focus much of the computational effort on these crucial parameters. The prioritization of parameters and time interval to calibrate them is expected to evolve as an area of importance, providing a direction to future *Omics *efforts in this area to provide systems-level measurements for virtually all types of cellular components and parameters in a model organism [33].

## Conclusion

In this investigation we incorporated the protein abundance information into our MRL framework to globally optimize elementary rate constants through a novel hybrid method. We effectively deal with the issues of network scale, stiffness, local minima, computational burden and parameter unidentifiability inherent within a large MRL model. Since the proposed method makes full use of the available experimental data, it addresses the problem of the computer simulations of biological systems which have high resolution regimes but lack experimental support at such a finer scale. The work presented here provides us with optimism that models in the mesoscopic regime (e.g. particle-based methods) can be rooted on a firm foundation of parameters generated in the macroscopic regime on an experimental basis.

Moreover, the resulting MRL models are as close as possible to the biological experiments. Therefore, they can be used to steer further biological experiments aimed at supporting computer simulation. For example, specific direction and guidance for sampling procedures can be issued after a time-dependent sensitivity analysis, through which the most sensitive parameters and time intervals are identified.

## Methods

**Figure 6:**
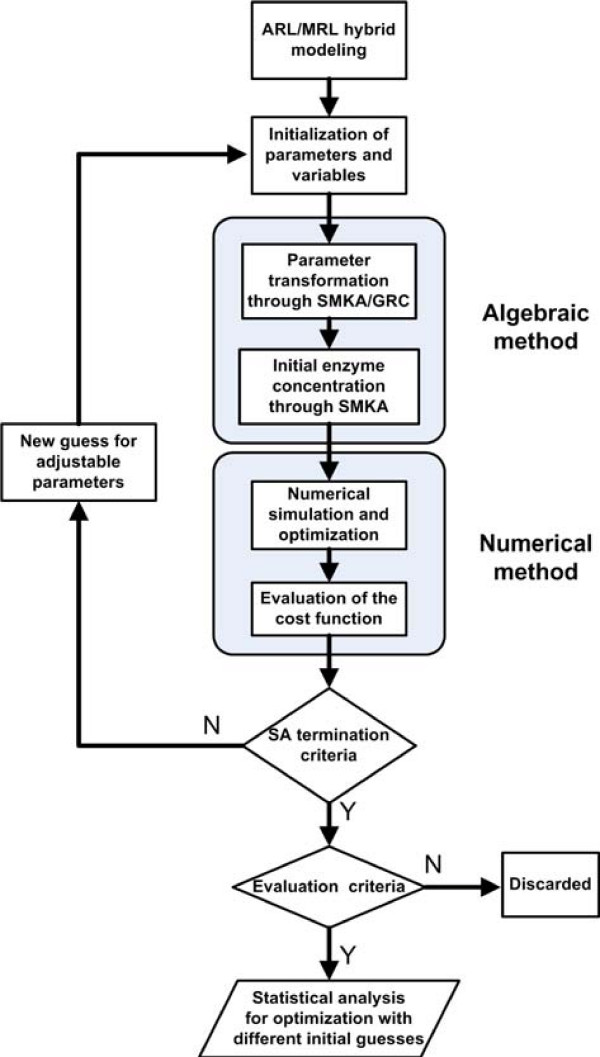
**A flow chart illustrating the process of extracting MRL constants from ARL models**. SMKA: Schematic Method of King and Altman; GRC: General Rule of Cleland; SA: simulated annealing algorithm.

The protocol for the extraction of MRL rate constants from ARL models follows a series of steps as shown in Figure 6, which are described in detail below.

### Hybrid model development

#### ARL and MRL

Before initiating the parameter optimization process, a kinetic model which will be the subject of the optimization has to be specified. This implies defining the ARL reactions, setting their MRL kinetic types, and identifying all of the variables and parameters. The ARL model, which is adapted from Chassagnole's dynamical model of *E. coli *central metabolism [21], consists of mass balance equations for extracellular glucose and for the intracellular metabolites as shown in Figure 1. The time course of unbalanced cometabolite (NAD+, NADH, NADP+, NADPH, AMP, ADP and ATP) concentrations were fitted with analytical functions [21]. Kinetic rate equations define the metabolic pathways through a coupled system of aggregated rate laws in the form of mechanistic or empirical rate expressions. The first step in transforming individual ARL reactions into mass-action form is to define the elementary steps of a reaction mechanism from a literature search on the individual enzymes; what remains is to find an appropriate set of parameters for the MRL model.

#### Hybrid MRL/ARL modelling

Direct replacement of every reaction step with its MRL mechanism leads to an unreasonably large number of parameters for simultaneous optimization. Therefore we have developed a hybrid modelling approach to optimize parameters for the MRL model. In this approach, the network is partitioned into modules, one for each enzymatic reaction. Then, a series of hybrid ARL/MRL models can be constructed, one for each ARL reaction, with that single individual ARL reaction replaced by its MRL version. Parameter optimization is done on the hybrid models, optimizing the parameters for that single set of MRL reactions alone in the context of the remaining ARL network. In this way, the number of simultaneous parameters requiring optimization is substantially reduced. An example is shown in Figure 1, illustrating the replacement of the phosphoglucoisomerase (*pgi*) pathway with the two individual reversible elementary reaction steps in the MRL form.

### Algebraic-numerical simulation and optimization

Once all the necessary information has been defined, it is then passed to the optimization module that combines SMKA/GRC with NSO.

#### Initial conditions

Initial concentrations of metabolites and cometabolites upon a glucose impulse are the same as for Chassagnole's ARL model [21] of *E. coli *growing on minimal medium. Initial enzyme concentrations shown in Table 1, except pyruvate kinase, are adapted from Lu et al. [34], where mass spectrometry-based absolute protein expression (APEX) profiling was developed for precise measurement of *E. coli *protein abundances. Protein abundance for pyruvate kinase is taken from 2-D gel data [35], since so far only abundance data from gel-based proteome technique is available. The adjustable parameters that are the subject of optimization need to be given an initial value as the optimization methods must start at some point of parameter space. For our proposed hybrid method, the adjustable parameters can take any initial value within boundary constraints that are detailed below. Adjustable parameters are selected based on one or more of the following criteria: (1) choose a parameter which can be classified as more sensitive than others through sensitivity analysis; (2) choose a parameter which has relatively small acceptable range; or (3) choose a parameter through which other rate constants can be determined. The selected adjustable parameters for each mechanism are defined in Additional File 1.

#### Algebraic manipulation

With given guesses for the adjustable MRL parameters, all MRL rate constants for an individual ARL reaction are estimated by applying SMKA/GRC, where combinations of MRL rate constants are algebraically related to known ARL kinetic constants. Then, given the enzyme concentration listed in Table 1, the initial steady-state concentration of all enzyme forms are determined from the SMKA distribution equations consisting of metabolite concentrations and estimated rate constants.

#### Numerical simulation and optimization

With algebra-derived MRL parameters from ARL constants, using the initial distribution of enzyme species, the dynamics of all of the elements and reaction rates upon glucose impulse were simulated in the hybrid ARL/MRL models using a stiff ODE solver (ode15s in Matlab). The resulting metabolite concentration and reaction rate curves were compared with the same curves generated using the ARL model alone, allowing the optimization process to find parameter values for the MRL steps which correspond as closely as possible to the ARL model.

The parameter optimization requires the automated comparison of multiple time-dependent curves for metabolite concentrations and reaction rates between the ARL and the MRL version. A mean-square error is a natural choice to measure the degree of similarity of hybrid model simulations to the original ARL model, but there is a certain degree of arbitrariness in the relative weightings of error terms in the cost function. We resolve this by weighting each error term by the peak value in the reference ARL time series, which effectively demands equal performance for all metabolites and reaction rates on their own scale [36]. The cost function is then

(1) $$fval=\sum_{n}^{NEC}\sum_{c}^{NTS}\sum_{t}^{NSP}{\left|X_{predicted}(n,c,t)-X_{reference}(n,c,t)\right|}^{2}*w(n,c)$$

where ***NEC ***is the Number of the Experimental Conditions for the data, ***NTS ***is the total Number of Time Series for metabolites and sub-step rates specific to an individual ARL reaction, ***NSP ***is the Number of Sampling Points in time series c, ***X*_*predicted*_*(n,c,t) ***is the Time Course Data from the hybrid model simulation, ***X*_*reference*_*(n,c,t) ***is the Time Course Data from the reference ARL simulation, and the weighting ***w(n,c) ***is $1/{\left|\underset{t}{\max}X_{reference}(n,c,t)\right|}^{2}$ for reference time series ***c ***at condition ***n***.

#### Generation of new guess

If the maximum iteration number for simulated annealing (i.e. the termination criterion) is not reached, a new guess for the adjustable parameters is generated. All MRL rate constants are then re-estimated by SMKA/GRC step and the cost function value is re-evaluated by numerical simulation. This cycle is repeated until satisfactory results are achieved. The evaluation is considered satisfactory if the evaluation criterion *fval *is less than 0.08. We have found that if *fval *is larger than 0.08, the MRL simulation results are unable to provide a reasonable match to the ARL simulation results.

#### Simulated annealing procedure [18]

The rate constants are globally optimized via the following simulated annealing procedure: the starting annealing temperature (T_0_) was set around the order of magnitude of the cost function at the initial estimates, and the annealing temperature was linearly decreased by a reduction factor (RF) of 0.1 until the temperature reached zero. In order to verify that the annealing schedule was able to explore the entire parameter space of the underlying MRL mechanism, multiple testing calculations were performed with varying RF around the preset value. The annealing schedule with the best cost function value is regarded as an optimal one for the global optimization process.

### Statistical analysis

#### Optimized parameters

A suitable number of optimizations (25 runs) were done with different random initial guesses distributed over the entire acceptable range of the rate constants. Mean and standard deviation (SD) of parameters were calculated from these 25 optimization runs. A 95% confidence interval (CI) for the fitted parameters and the coefficient of variation (CV = SD/Mean) were used to evaluate the precision and variation of the parameters.

#### Effect of experimental uncertainty in enzyme level

The noise was computer generated with random numbers based on Gaussian distribution and added to the experimentally determined value for *pykF *(1.2 μM, which was measured by 2-D gel with some uncertainty). Twenty-five noisy enzyme concentrations were randomly generated for each noise level (10% and 20%) and the optimization was repeated. A 95% confidence interval (CI) for the fitted parameters and the relative errors (RE) between noise and 'noise-free' solutions were used to evaluate the parameter precision and bias due to experimental uncertainty.

### Example of a multisubstrate kinetic mechanism

Glyceraldehyde 3-phosphate dehydrogenase (*gapA*), which obeys a multisubstrate kinetic mechanism, is used as an example to illustrate and explain the basic steps of the hybrid algebraic-numerical method.

Chassagnole's model for *gapA *is a simplified ARL equation, since neither the sequence of subreactions nor the enzyme forms can be deduced from the model. The first binding of NAD^+ ^to the enzyme and the last release of NADH from the enzyme have been found for NAD^+^-linked dehydrogenases [37]. So an ordered sequential mechanism would be expected with *gapA*; what remains is to identify if the binding mechanism proceeds through a ternary complex (Figure 4A) or through a binary complex (Figure 4B). There is experimental evidence that a kinetically significant ternary complex exists for NAD+-linked dehydrogenases [37], so the optimization process presented here starts with an ordered Bi Bi mechanism with a ternary central complex. Treating *k*_-2 _and *k*_4 _as adjustable parameters leads to a set of equality constraints for rate constants and enzyme forms (See Additional file 1 for details). By varying *k*_-2 _and *k*_4_, the optimization process automatically tries to arrive at the best solution for MRL parameters, so that the resulting concentration and rate curves correspond as closely as possible to the same curves generated using the ARL model alone. In the cost function (Eqn. 1), *NEC *is 1, relating to a glucose impulse to extracellular concentration of 2 mM. *NTS *is 6, consisting of 2 time-series for metabolites and 4 time-series for net rates of four sub-steps. *NSP *is 21, relating to a sampling time interval of 0–20 s with a sampling point every 1 s. The time period over which we run the simulation and optimization is consistent with the original experiment, where all the intracellular metabolites could be sampled and measured within 20 seconds after the glucose impulse [21].

### Example for allosteric regulation

Pyruvate kinase (*pykF*) is an allosteric enzyme whose kinetic behaviour is usually described by the concerted allosteric transition mode of the Monod, Wyman, and Changeux (MWC) model [38]. According to the MWC model, *pykF *can exist in an active state (ER) or an inactive state (ET). The fraction of active enzyme in the ER or ET states is determined by the concentrations and relative affinities of the inhibitor (ATP) and the activator (FDP) for the ER and ET states [39]. In addition to this allosteric regulation, the enzyme normally follows the substrates binding rule in the order PEP and ADP and the products releasing rule in the order PYR and ATP [24]. Since the equilibrium constant of the *pykF *reaction is much larger, of the order of 10^5 ^[40], this reaction is always regarded as irreversible with the back reaction being ignored completely.

Assuming that the reaction within the allosteric segment (dark shaded area as shown in Figure 2A) reaches near equilibrium, the Cha method [41] can be used for the derivation of the complete King-Altman equations by considering all the enzyme forms within the allosteric segment as a single entity, i.e. as a single corner of the basic King-Altman figure. We can call the allosteric segment 'ETER', which consists of active and inactive states of the enzyme. The symbol *f*, representing *fractional concentrations *[41], is introduced in the sequential multisubstrate reactions to stand for the relative proportion of the equilibrium segment, ETER, that actually is involved in the given sequential multisubstrate reactions. For example, considering the species constituting ETER in Figure 2A, it is ER that reacts with PEP (with a rate coefficient *k*_1_) to yield ER-PEP. The symbol *f *represents the proportion of ETER that is ER, which is obtained by the usual rules for equilibrium systems [41]

(2) $$f=\frac{\left[ER\right]}{\left[ETER\right]}=\frac{\frac{k_{-5}}{k_{5}}\cdot {\left(\frac{\left[FDP\right]}{\left[ATP\right]}\right)}^{4}}{1+\frac{k_{-5}}{k_{5}}\cdot {\left(\frac{\left[FDP\right]}{\left[ATP\right]}\right)}^{4}}=\frac{\frac{1}{K_{eq}}\cdot {\left(\frac{\left[FDP\right]}{\left[ATP\right]}\right)}^{4}}{1+\frac{1}{K_{eq}}\cdot {\left(\frac{\left[FDP\right]}{\left[ATP\right]}\right)}^{4}}$$

where *K*_*eq *_is the equilibrium constant and the power of 4 is the number of the allosteric sites for *pykF *[39]. Thus, *k*_1_*·f *in Figure 2A represents an effective rate constant for the first step of the sequential multisubstrate reactions, and the SMKA/GRC treatments yields all the enzyme forms and rate constants as shown in Additional file 1.

### MRL model evaluation

#### Parameter sensitivity

We performed a time-dependent sensitivity analysis in order to determine the effects of rate constants on time series of glycolytic rates. We define the scaled sensitivities for time-dependent fluxes of reactions by

(3) $$S(J_{i}(t),p_{j})=\frac{p_{j}}{J_{i}(t)}\cdot \frac{\partial J_{i}(t)}{\partial p_{j}}=\frac{\partial \log J_{i}(t)}{\partial \log p_{j}}$$

where *J*_*i*_*(t) *are the time course of glycolytic rates and *p*_*j *_are rate constants. Eqn. (3) is evaluated by numerical differentiation of the network model using finite differences.

#### Coupled-enzyme system

To evaluate the performance of the MRL model for the coupled-enzyme system, we simultaneously replaced ARL pathways corresponding to glycolysis (9 separate ARL reactions) with their MRL versions in the context of the remaining ARL model. For each enzyme, we picked the set of the rate constants with the best cost function values.

The validity of the MRL assembly was tested by comparing transient dynamic responses after a glucose impulse with those of the original ARL network. The stability of the model was evaluated by the eigenvalues of the Jacobian matrix (A). While A is a complete 18 × 18 matrix for the ARL network of 18 species, a reduced 36 × 36 Jacobian matrix can be implemented for the MRL network of 45 species, due to the removal of 9 dependent species as a result of the conservation relations for enzyme forms. The Jacobian matrix (A) at the steady state can be calculated for an n-dimensional dynamical system, where *x *is a vector of species [*x*_1_, *x*_2_, ... *x*_*n*_] and $\frac{dx}{dt}=f(x)$, as

(4) $$A=\left(\begin{array}{ccc} \frac{\partial f_{1}}{\partial x_{1}} & \cdots & \frac{\partial f_{1}}{\partial x_{n}}\\ \vdots & \ddots & \vdots \\ \frac{\partial f_{n}}{\partial x_{1}} & \cdots & \frac{\partial f_{n}}{\partial x_{n}} \end{array}\right)$$

The eigenvalues *u *satisfy the characteristic equation of the matrix A

(5) det(*A *- *ul*) = 0

Eqn. (5) is a polynomial equation in *u *of degree n, and *I *is the identity matrix. By the Fundamental Theorem of Algebra, this equation has n solutions. If all the eigenvalues of A have negative real part then the steady states of all species are stable. The time constant, which indicates how fast a deviation from a given steady state will decline, is defined as the reciprocal absolute values of the real parts of the eigenvalues [42]. In addition, the stiffness or eigenvalue spread of the model can be calculated as the ratio of the largest over the smallest eigenvalue.

### Simplications, Boundary constraints, and Tools

#### Simplications for Phosphofructokinase

Allosterism can cause fractal properties [43] and kinetic changes due to enzyme conformational changes. To avoid the vast expansion of parameters to be optimized, in this situation we model the particularly complicated allosteric regulation for phosphofructokinase (*pfkA*) via non-integral reaction orders, thereby breaking from the pure elementary reaction schema, but maintaining the type of bimolecular reactions (Figure 2B). This treatment for its simplicity greatly increased the ability to characterize reaction parameters. Since the original ARL model is almost an empirical expression for *pfkA *dynamics, it is impossible to establish a relationship between kinetic constants and rate constants through SMKA/GRC. For this reason, we treat all rate constants and non-integral reaction orders as adjustable parameters that vary freely within the acceptable ranges that are detailed below. These parameters were used to calculate the initial concentrations of enzyme forms through SMKA (see Additional file 1) and then estimated by numerical simulation and optimization to reproduce the original time-course data for the ARL model.

#### Boundary constraints

When two reactants for bimolecular reactions are approximately equal in size, the maximum diffusion-limited association rate constant for molecular interactions corresponds to *k*_*a *_≈ 10^6^-10^7 ^mM^-1 ^s^-1^. Since the association could be even faster for enzymatic reactions where one molecule is small and diffuses rapidly while the other is large and provides a large target [44], the order of magnitude for association rate constants are constrained to be not over 10^9 ^mM^-1 ^s^-1^. The dissociation rate constants are required to not be in excess of 10^6 ^s^-1 ^[45].

Parameters for phosphofructokinase (*pfkA*) are confined within a relatively small parameter space, which is based on mechanistic analysis and time-consuming trial-and-error testing. Bacterial *pfkA *enzyme has been found to be inhibited by PEP as a result of interaction at an allosteric site [46] and also by a high concentration of ATP due to substrate antagonism [47]. Considering the high levels of PEP and ATP in this study, it is reasonable to assume that the involved elementary steps have low rate constants, except for the last release of ADP where a higher rate constant is necessary for ensuring the fast breakdown of ER-ADP in order to provide as much as possible of the enzyme in the active state (Figure 2B). For these reasons, the upper boundaries of the forward rate constants are set at 1000 mM^-1 ^s^-1^, 5000 mM^-1 ^s^-1^, 1000 s^-1 ^and 1000000 s^-1 ^for *k*_1_, *k*_2_, *k*_3 _and *k*_4_, respectively, and the backward rate constants are confined within 100 s^-1 ^and 2000 s^-1 ^for *k*_-1 _and *k*_-2_, respectively. The upper boundaries were further verified by random testing, where parameters out of the preset ranges appeared unable to reach a satisfactory cost function value.

#### Tools

Matlab (Mathworks, Nattick MA) toolboxes [48] were used for model simulation and optimization, with models compiled into MEX binaries for performance [49]. The simulated annealing (SA) code was partly based on the C code of Press and Teukolsky [50].

## Abbreviations

The following abbreviations are used for individual enzymes: ***aroH ***– DAHP synthase (EC 2.5.1.54), ***eno ***– Enolase (EC 4.2.1.11), ***fba ***– Fructose-bisphosphate aldolase (EC 4.1.2.13), ***gapA ***– Glyceraldehyde 3-phosphate dehydrogenase (EC 1.2.1.12), ***glgC ***– Glucose-1-phosphate adenylyltransferase (EC 2.7.7.27), ***gnd ***- 6-phosphogluconate dehydrogenase (EC 1.1.1.44), ***gpsA ***– Glycerol 3-phosphate dehydrogenase (EC 1.1.1.94), ***pdhA ***– Pyruvate dehydrogenase (EC 1.2.4.1), ***pfkA ***– Phosphofructokinase (EC 2.7.1.11), ***pgi ***– Phosphoglucoisomerase (EC 5.3.1.9), ***pgk ***– Phosphoglycerate kinase (EC 2.7.2.3), ***pgm ***– Phosphoglycerate mutase (EC 5.4.2.1), ***pgm1 ***– Phosphoglucomutase (EC 5.4.2.2), ***ppc ***– Phosphoenolpyruvate carboxylase (EC 4.1.1.31), ***prs ***– Ribose phosphate pyrophosphokinase (EC 2.7.6.1), ***pykF ***– Pyruvate kinase (EC 2.7.1.40), ***rpe ***– Ribulose phosphate epimerase (EC 5.1.3.22), ***rpi ***– Ribose phosphate isomerase (EC 5.3.1.6), ***talA ***– Transaldolose (EC 2.2.1.2), ***tktA ***– Transketolase a (EC 2.2.1.1), ***tktB ***– Transketolose b (EC 2.2.1.1), ***tpiA ***– Triosephosphate isomerase (EC 5.3.3.1), ***zwf ***– Glucose-6-phosphate dehydrogenase (EC 1.1.1.49). The following abbreviations are used for metabolites: **DHAP **– Dihydroxyacetonephosphate, **E4P **– Erythrose-4-phosphate, **F6P **– Fructose-6-phosphate, **FDP **– Fructose-1,6-bisphosphate, **G1P **– Glucose-1-phosphate, **G6P **– Glucos-6-phosphate, **GAP **– Glyceraldehyde-3-phosphate, **GLC **– Glucose, **PEP **– Phosphoenolpyruvate, **PG **– 6-phosphogluconate, **PG2 **– 2-phosphoglycerate, **PG3 **– 3-phosphoglycerate, **PGP **– 1,3-diphosphoglycerate, **PYR **– Pyruvate, **Rib5P **– Ribose-5-phosphate, **Ribu5P **– Ribulose-5-phosphate, **Sed7P **– Sedoheptulose-7-phosphate, **Xyl5P **– Xylulose-5-phosphate. Other abbreviations are: **ARL **– Aggregated Rate Law, **CI **– Confidence Interval, **CV **– Coefficient of Variation, ***fval ***– Cost Function Value, **GRC **– General Rules of Cleland, **MEX **– MATLAB Executable Files, **MRL **– Mass Action Rate Law, **NSO **– Numerical Simulation and Optimization, **ODE **– Ordinary Differential Equation, **PBS **– Particle Based Simulation, **QSSA **– Quasi Stationary State Assumption, **RE **– Relative Errors compared to 0% noise case, **SD **– Standard Deviation, **SMKA **– Schematic Method of King and Altman.

## Authors' contributions

JZ and ME conceived and designed experiments. JZ developed computer programs and performed the numerical experiments. JZ and DR analyzed the data. JZ, DR, GB, AK and ME wrote the paper.

## Supplementary Material

Additional file 1 Additional file 1 (3 pages, see main manuscript for abbreviations and elementary reaction steps). A.1. Ordered Bi-Bi mechanism for *gapA*. A.2. Allosteric regulation for *pykF*. A.3. Allosteric regulation for *pfkA*. A.4. Ordered Uni-Bi mechanism for *fba*A.5. Reversible Uni-Uni mechanism for *pgi* Click here for file
