# Supplementary material for: Extraction of elementary rate constants from global network analysis of E. coli central metabolism
Source: BMC Syst Biol. 2008 May 7;2:41. doi: 10.1186/1752-0509-2-41 (PMC2396597; doi:10.1186/1752-0509-2-41)
Supplement: Additional file 1 — (3 pages, see main manuscript for abbreviations and elementary reaction steps). A.1. Ordered Bi-Bi mechanism for gapA. A.2. Allosteric regulation for pykF. A.3. Allosteric regulation for pfkA. A.4. Ordered Uni-Bi mechanism for fbaA.5. Reversible Uni-Uni mechanism for pgi [file 1752-0509-2-41-S1.pdf]

Additional file 1

A.1. Ordered Bi-Bi mechanism for *gapA*

By treating k_{-2} and k_4 as adjustable parameters to be optimized, the SMKA/GRC method yields a set of equations which will be used as inputs at the beginning of each numerical iteration

$$k1 = Vmax/[E]_{total} / K_{m,\ nad} \quad (1)$$

$$k\_1 = k1*K_{i,\ nad} \quad (2)$$

$$k3 = (k4*Vmax/[E]_{total})/(k4-Vmax/[E]_{total}) \quad (3)$$

$$k2 = (k4*(k\_2+k3))/((k3+k4)* K_{m,\ gap}) \quad (4)$$

$$k\_3 = (k\_1*(k\_2+k3))/(K_{m,\ pgp}*(k\_1+k\_2)) \quad (5)$$

$$k\_4 = (k1*k2*k3*k4)/(k\_1*k\_2*k\_3*KEQ) \quad (6)$$

$$[E] = ((k4*k\_1*k\_2+k\_3*cpgp*k\_2*k\_1+k2*cgap*k3*k4+k\_1*k3*k4)/ Denominator)*[E]_{total} \quad (7)$$

$$[E\text{-}NAD] = ((k4*k1*cnad*k\_2 + k1*cnad*k\_3*cpgp*k\_2 + k\_4*cnadh*k\_3*cpgp*k\_2 + k3*k4*k1*cnad)/ Denominator)*[E]_{total} \quad (8)$$

$$[E\text{-}NAD\text{-}GAP] = ((k4*k1*cnad*k2*cgap + k1*cnad*k2*cgap*k\_3*cpgp + k\_4*cnadh*k\_3*cpgp*k2*cgap + k\_1*k\_4*cnadh*k\_3*cpgp) / Denominator)*[E]_{total} \quad (9)$$

$$[E\text{-}NADH] = ((k\_2*k\_1*k\_4*cnadh + k1*cnad*k2*cgap*k3 + k\_4*cnadh*k2*cgap*k3 + k\_1*k\_4*cnadh*k3) / Denominator)*[E]_{total} \quad (10)$$

where Denominator=k_1*k4*(k_2+k3)+k1*k4*(k_2+k3)*cnad+k2*k3*k4*cgap+k_1*k_2*k_3*cpgp + k_1*k_4*(k_2 + k3)*cnadh + k1*k2*(k3 + k4)*cnad*cgap + k1*k_2*k_3*cnad*cpgp + k_3*k_4*(k_1 + k_2)*cpgp*cnadh + k2*k3*k_4*cgap*cnadh + k1*k2*k_3*cnad*cgap*cpgp + k2*k_3*k_4*cgap*cpgp*cnadh (11)

A.2. Allosteric regulation for *pykF*

The enzyme *pykF* supports a sequential-ordered Bi-Bi mechanism for two substrates, while possessing separate allosteric sites for allosteric activators and inhibitors. By treating k_{-1}, k_{-2} and k_3 as adjustable parameters to be optimized, the SMKA/GRC treatments yields a set of equations for other rate constants and all the enzyme forms

$$k1= (Vmax/[E]_{total})/K_{m,\ pep}/f \quad (12)$$

$$k4=1/([E]_{total} /Vmax-1/k3) \quad (13)$$

k2=(Vmax/$[E]_{total}$ /$K_{m, adp}$)*((k_2+k3)/k3) (14)

[ETER]= ((k4*k_1*k_2+k2*cadp*k3*k4+k_1*k3*k4)/ Denominator) *$[E]_{total}$ (15)

[ER-PEP]= ((k4*k1**f**cpep*k_2+k3*k4*k1**f**cpep)/ Denominator)*$[E]_{total}$ (16)

[ER-PEP-ADP]= ((k4*k1**f**A*k2*cadp)/ Denominator)*$[E]_{total}$ (17)

[ER-ATP]= ((k1**f**A*k2*cadp*k3)/ Denominator)*$[E]_{total}$ (18)

where Denominator = (k4*k_1*k_2 + k2*cadp*k3*k4 + k_1*k3*k4 + k4*k1**f**cpep*k_2 + k3*k4*k1**f**cpep+ k4*k1**f**cpep*k2*cadp+k1**f**cpep*k2*cadp*k3)

(19)

A.3. Allosteric regulation for *pfkA*

Since the original ARL model is almost an empirical expression for *pfkA* dynamics, it is impossible to establish a relationship between kinetic constants and rate constants through SMKA/GRC. For this reason, we treat all rate constants and non-integral reaction orders as adjustable parameters freely varying within the acceptable ranges. These parameters were used to calculate the initial concentrations of enzyme forms through SMKA

Denominator = (k4*k_1*k_2 + k2*cf6p^n*k3*k4 + k_1*k3*k4 + k4*k1*f*catp^m*k_2 + k3*k4*k1*f*catp^m + k4*k1*f*catp^m*k2*cf6p^n + k1*f*catp^m*k2* cf6p^n*k3)

(20)

ETER = ((k4*k_1*k_2+k2*cf6p^n*k3*k4+k_1*k3*k4)/Denominator)*$[E]_{total}$ (21)

ER-ATP = ((k4*k1*f*catp^m*k_2+k3*k4*k1*f*catp^m)/Denominator)*$[E]_{total}$ (22)

ER-ATP-F6P = ((k4*k1*f*catp^m*k2*cf6p^n)/Denominator)*$[E]_{total}$ (23)

ER-ADP = ((k1*f*catp^m*k2*cf6p^n*k3)/Denominator)*$[E]_{total}$ (24)

A.4. Ordered Uni-Bi mechanism for *fba*

By treating k_{-1} as adjustable parameters to be optimized, the SMKA/GRC method yields a set of equations which will be used as inputs at the beginning of each numerical iteration

k_3 = k_1*VF/VC (25)

k3 = (VB/VD)*k_3 (26)

k1 = k_3/(VE*VF) (27)

k2 = (VA*k3)/(k3-VA) (28)

$k\_2 = (k2 + k3)/VE$ (29)

Where

$VA = V_{max, forward} / [E]_{total}$ (30)

$VB = K_{m, fdp}$ (31)

$VC = K_{m, fdp}/KEQ/VALDOBLF$ (32)

$VD = K_{m, gap}/KEQ/VALDOBLF$ (33)

$VE = K_{i, gap}$ (34)

$VF = 1/KEQ/VALDOBLF$ (35)

and VALDOBLF is the ratio of $V_{max, forward}$ to $V_{max, backward}$.

SMKA then yields the distribution equations for all enzyme forms at time 0, which will be used as inputs at the beginning of each numerical iteration.

Denominator = (k_3*k_2*cdhap*cgap + k1*k_2*cfdp*cgap + (k_1*k_3+k2*k_3)*cdhap + k_1*k_2*cgap + (k1*k3 + k1*k2)*cfdp + (k_1*k3 + k2*k3) (36)

EA = ((k1*k_2*cfdp*cgap + k_3*k_2*cdhap*cgap + k3*k1*cfdp)/Denominator)*$[E]_{total}$ (37)

E = ((k_1*k3 + k3*k2 + k_2*k_1*cgap)/ Denominator)*$[E]_{total}$ (38)

EQ = ((k1*k2*cfdp + k_1*k_3*cdhap + k_3*k2*cdhap)/Denominator)*$[E]_{total}$ (39)

A.5. Reversible Uni-Uni mechanism for *pgi*

By treating k_{-2} as adjustable parameters to be optimized, the SMKA/GRC method yields a set of equations which will be used as inputs at the beginning of each numerical iteration

k2=Vmax/$[E]_{total}$ (40)

k_1=k2/(KEQ*($K_{m, g6p}$ / $K_{m, f6p}$)) (41)

k1=(k_1+k2)/ $K_{m, g6p}$ (42)

EA=((k1*cg6p+k_2*cf6p)/ Denominator)*$[E]_{total}$ (43)

E=((k_1+k2)/ Denominator)*$[E]_{total}$ (44)

Where Denominator = (k1*cg6p+k_2*cf6p+k_1+k2) (45)
